# 1.5T Magnetic Resonance-Guided Stereotactic Body Radiotherapy for Localized Prostate Cancer: Preliminary Clinical Results of Clinician- and Patient-Reported Outcomes

**DOI:** 10.3390/cancers13194866

**Published:** 2021-09-28

**Authors:** Darren M. C. Poon, Jing Yuan, Oi-Lei Wong, Bin Yang, Sin-Ting Chiu, Kin-Yin Cheung, George Chiu, Siu-Ki Yu

**Affiliations:** 1 Comprehensive Oncology Centre, Hong Kong Sanatorium & Hospital, Happy Valley, Hong Kong, China; 2 Medical Physics and Research Department, Hong Kong Sanatorium & Hospital, Happy Valley, Hong Kong, China; jyuanbwh@gmail.com (J.Y.); penguin.oilei@gmail.com (O.-L.W.); kimi.b.yang@gmail.com (B.Y.); KinYin.Cheung@hksh.com (K.-Y.C.); benyu@hksh.com (S.-K.Y.); 3 Department of Radiotherapy, Hong Kong Sanatorium & Hospital, Happy Valley, Hong Kong, China; SinTing.Chiu@hksh.com (S.-T.C.); george.chiu@hksh.com (G.C.)

**Keywords:** Expanded Prostate Cancer Index Composite (EPIC), image-guided radiation therapy, magnetic resonance-guided radiotherapy (MRgRT), patient-reported outcome measures (PROMs), prostate cancer, questionnaire, stereotactic body radiotherapy (SBRT), toxicity

## Abstract

**Simple Summary:**

The combination of stereotactic body radiotherapy (SBRT) and magnetic resonance-guided radiation therapy (MRgRT) offers the potential for achieving better tumor control and lower toxicities for prostate cancer (PC) patients. This study reports for the first time preliminary longitudinal clinical results of 1.5T MR-guided SBRT (MRgSBRT) in a cohort of 51 localized PC patients (median follow-up: 199 days; range: 41–424 days), based on both clinician-reported outcome measurement (CROM) and patient-reported outcome measurement (PROM). The maximum cumulative clinician-reported grade ≥ 2 (Common Terminology Criteria for Adverse Events Scale v. 5.0) acute genitourinary (GU) and gastrointestinal (GI) toxicities were 11.8% (6/51) and 2.0% (1/51), respectively, while grade ≥ 2 subacute GU and GI toxicities were 2.3% (1/43) each. Patient-reported urinary, bowel, and hormonal domain summary scores were reduced at 1 month, then gradually returned to baseline levels, with the exception of the sexual domain. The finding of low toxicity supports the accumulation of clinical evidence for 1.5T MRgSBRT in localized PC.

**Abstract:**

Background: Magnetic resonance-guided stereotactic body radiotherapy (MRgSBRT) offers the potential for achieving better prostate cancer (PC) treatment outcomes. This study reports the preliminary clinical results of 1.5T MRgSBRT in localized PC, based on both clinician-reported outcome measurement (CROM) and patient-reported outcome measurement (PROM). Methods: Fifty-one consecutive localized PC patients were prospectively enrolled with a median follow-up of 199 days. MRgSBRT was delivered in five fractions of 7.25–8 Gy with daily online adaptation. Clinician-reported gastrointestinal (GI) and genitourinary (GU) adverse events based on the Common Terminology Criteria for Adverse Events (CTCAE) Scale v. 5.0 were assessed. The Expanded Prostate Cancer Index Composite Questionnaire was collected at baseline, 1 month, and every 3 months thereafter. Serial prostate-specific antigen measurements were longitudinally recorded. Results: The maximum cumulative clinician-reported grade ≥ 2 acute GU and GI toxicities were 11.8% (6/51) and 2.0% (1/51), respectively, while grade ≥ 2 subacute GU and GI toxicities were 2.3% (1/43) each. Patient-reported urinary, bowel, and hormonal domain summary scores were reduced at 1 month, then gradually returned to baseline levels, with the exception of the sexual domain. Domain-specific subscale scores showed similar longitudinal changes. All patients had early post-MRgSBRT biochemical responses. Conclusions: The finding of low toxicity supports the accumulation of clinical evidence for 1.5T MRgSBRT in localized PC.

## 1. Introduction

Prostate cancer (PC) is the second most common type of cancer among males behind lung cancer, accounting for ~3.8% of all deaths in men in 2018 [1]. In the management of PC, active surveillance has become the preferred approach for men with less-aggressive disease. Surgery and radiotherapy (RT) continue to be curative treatments for localized PC, but have different patterns of adverse effects that can negatively affect quality of life (QoL) [2].

Moderate hypofractionation has been shown to be non-inferior to conventional fractionation in RT for localized PC in a number of clinical trials [3,4,5,6]. Furthermore, the clinical use of ultra-hypofractionation with a dose/fraction of 5.0 Gray (Gy) or higher, also known as stereotactic body RT (SBRT), has also been actively explored in localized PC RT in recent years [7,8,9,10,11]. The PACE-B phase 3 trial suggested that SBRT (36.25 Gy in 5 fractions) did not increase either gastrointestinal (GI) or genitourinary (GU) acute toxicity for up to 12 weeks after RT in low/intermediate-risk PC patients [11]. The HYPO-RT-PC phase 3 trial showed that ultra-hypofractionation (42.7 Gy in seven fractions, 3 days/week) was non-inferior to conventionally fractionated RT for intermediate/high-risk PC patients in terms of failure-free survival, although clinician-reported early side effects were more pronounced [10]. The long-term patient-reported QoL analysis of HYPO-RT-PC data showed no significant difference in the incidence of clinically relevant deterioration between the two groups for overall urinary/bowel/sexual bother or global health/QoL. This finding suggested that ultra-hypofractionation was as well tolerated as conventional fractionation for up to 6 years after treatment [9]. SBRT is now considered to be an alternative to conventionally fractionated RT in patients with low/intermediate risk PC [5].

Magnetic resonance-guided RT (MRgRT) [12,13,14,15] is an innovative technique to guide SBRT, offering potential advantages over the existing SBRT guidance techniques. Systems that integrate MR imaging (MRI) with a linear accelerator (MR-Linac) [16,17,18] are used clinically [14], enabling superior soft-tissue image contrast without ionizing radiation. They offer better visualization of on-the-day anatomy for online treatment adaptation, eliminating the need for invasive fiducial marker implantation and improving margin reduction capability [19]. This distinctive feature of MRgRT holds the potential to achieve better tumor control and lower toxicities of PC SBRT. However, reports of clinical outcomes of MR-guided SBRT (MRgSBRT) in PC remain sparse [20,21,22,23]. The longitudinal clinical outcome of 1.5T MRgSBRT in localized PC is not yet available. Thus, the major purpose of this longitudinal observational study from the real world was to report the preliminary results of MRgSBRT in localized PC conducted on a 1.5T MR-Linac (Unity, Elekta, Stockholm, Sweden) from a single center, based on both clinician-reported outcome measurement (CROM) and patient-reported outcome measurement (PROM) data.

## 2. Results

### 2.1. Patient Selection and Baseline Characteristics

Between March 2020 and June 2021, 56 localized PC patients underwent 1.5T MRgSBRT treatment; five patients were excluded due to missing baseline or follow-up PROM data. Finally, 51 consecutive male patients (age, 71.5 ± 7.7 years; range, 56–90 years) were included. Patient and tumor characteristics at the pre-treatment baseline are summarized in Table 1. Thirty patients were prescribed androgen deprivation therapy (ADT).

### 2.2. Treatment Delivery and Adaptation

All treatment fractions were successfully delivered to the 51 patients without interruption. Of the total 255 (51 × 5) fractions, adapt-to-position (ATP) and adapt-to-shape (ATS) workflows were used for online daily treatment adaptation in 29 (11.4%) and 226 fractions (88.6%), respectively. The average in-room time for daily adaptive treatment procedures, including the steps of patient positioning, daily MR imaging, recontouring, plan adaptation, quality assurance, and dose delivery, was 49 min for ATP and 78 min for ATS. The average beam-on time was ~18 min.

### 2.3. CROMs

The clinician-reported GI and GU adverse events (AEs) are summarized in Table 2. Thirteen acute grade 1 GI AEs comprised two (3.9%) abdominal pain, one (2.0%) constipation, three (5.9%) diarrhea, one (2.0%) fecal incontinence, one nausea (2.0%), four (7.8%) proctitis, and one rectal pain (2.0%), in 10 patients (n = 51). No grade 2, but one grade 3 AE (2.0%) of proctitis was observed. At longer follow-ups, five GI-adverse events were reported (n = 43), including one grade 1 constipation (2.3%), two grade 1 proctitis (4.7%), and one of each grade 1 and grade 2 rectal hemorrhage (2.3% each).

More GU AEs were reported than GI AEs, with 11.8% (n = 6) of all patients having grade ≥ 2 acute GU AEs. Urinary frequency was most frequently reported (n = 5, 9.8%), followed by urinary tract pain (n = 3, 5.9%), urinary retention (n = 2, 3.9%), and urinary urgency (n = 1, 2.0%). Only one grade ≥ 2 subacute AE of urinary retention (n = 1, 2.3%) was reported. The proportion of patients with grade 1 GU AEs greatly increased from 47.1% at baseline to 76.5% within 30 days of MRgSBRT and, then, decreased to 58.1% at longer follow-ups.

**Figure 1:**
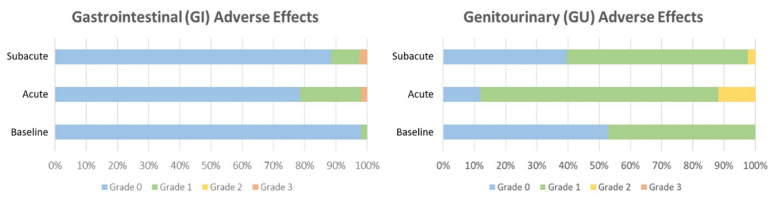
Clinician-reported genitourinary (GU) and gastrointestinal (GI) adverse effects based on the Common Terminology Criteria for Adverse Events Scale (CTCAE) v. 5.0 at different time points. Baseline: before the start of magnetic resonance-guided stereotactic body radiotherapy (MRgSBRT); acute, during MRgSBRT and ≤30 days after the completion of MRgSBRT; subacute, >30 days after the completion of MRgSBRT until the last follow-up.

The maximum cumulative incidence for any symptom was 11.8% for grade ≥ 2 acute GU AEs, and 2.0% for grade ≥ 2 acute GI AEs. Most other grade 2 GI and GU AEs were resolved at longer follow-ups. No additional grade ≥ 2 AEs developed in any patient at longer follow-ups. Figure 1 illustrates the proportion of GI and GU AEs at different time points.

### 2.4. PROMs

Patient follow-ups following completion of the last MRgRT fraction ranged from 41 to 424 days (median, 199 days). PROMs assessed by the Expanded Prostate Cancer Index Composite (EPIC) Questionnaire are summarized in Table 3.

**Figure 2:**
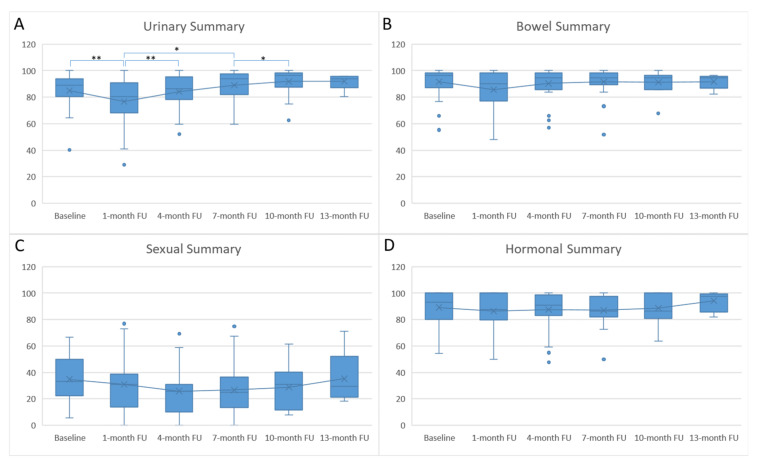
Boxplots of the Expanded Prostate Cancer Index Composite Questionnaire results. (**A**) Urinary, (**B**) bowel, (**C**) sexual, and (**D**) hormonal domain summary scores with regard to follow-up (FU) time. The cross point (×) indicates mean value. The line inside each bar indicates median value. Statistically significant differences between each two time points (Mann–Whitney U test) are indicated by * (*p* < 0.05) and ** (*p* < 0.01).

At 1 month, summary scores of all four domains (urinary, bowel, sexual, and hormonal) were reduced from the baseline values. After that, the scores increased, returning to baseline levels at 4 months and, then, remained relatively stable except for the sexual domain. Sexual domain scores were much lower than baseline until 13 months. However, only the urinary domain scores showed significant differences across the entire follow-up period (*p* = 0.0028, one-way analysis of variance [ANOVA]). Boxplots of the four domain summary scores from baseline through follow-up are shown in Figure 2.

Regarding the domain-specific subscales, scores generally exhibited similar trends as the domain summary scores, decreasing at the 1-month follow-up and recovering thereafter. Three urinary subscale scores, including function, bother, and irritative/obstructive, showed significantly different scores during follow-ups, while other subscales showed non-significant differences.

### 2.5. Early Prostate-Specific Antigen (PSA) Response

PSA levels were reduced from a baseline of 12.74 ± 8.15 ng/mL to 1.56 ± 2.07, 0.92 ± 1.41, 0.79 ± 1.10, 0.67 ± 1.14, and 0.51 ± 0.40 ng/mL at the 1-month, 4-month, 7-month, 10-month, and 13-month follow-ups, respectively. No patient developed biochemical recurrence using the Phoenix definition (PSA nadir + 2 ng/mL).

## 3. Discussion

The 1.5T MR-Linac offers a promising treatment modality for PC SBRT, postulated to further improve tumor control and reduce toxicity. To our knowledge, this is the first study to report the longitudinal clinical outcomes of 1.5T MRgSBRT in localized PC. The findings in this study, although preliminary, should be helpful to assess the clinical value of 1.5T MRgSBRT in these patients.

Several single-center studies have reported clinical outcomes of localized PC MRgSBRT in recent years. Researchers in Amsterdam reported 3-month early toxicity results [20] and the final 1-year follow-up PROM results [21] of a single-institute prospective single-arm phase 2 study conducted on a 0.35T MR-Linac (MRIdian system, ViewRay). Among their 101 patients, the largest treatment effects on urinary and bowel symptoms were recorded in the first 6-week follow-up. Thereafter, all symptoms decreased and returned to baseline values at 12 months, according to both PROMs and CROMs. Cumulative grade ≥ 2 GU and GI toxicities of 23.8% and 5.0%, respectively, at 3 months were reported [20]. The final 1-year result showed grade ≥ 2 GU toxicity between 3.1% and 5.1%, with no grade ≥ 2 GI toxicity [21]. Ugurluer et al. [22] also reported 0.35T MRgSBRT results in 50 patients followed for 3–29 months: grade 2 acute (during MRgSBRT) GU and GI rates were 36% and 0, respectively. At the 29-month mark, 2% and 6% patients experienced grade 2 GU and GI toxicities, respectively. Recently, Alongi et al. [23] presented the first preliminary report on feasibility, QoL, and PROMs in 25 localized PC patients treated with 1.5T MRgSBRT. No grade 3 AEs were observed, while three patients (12%) had grade 2 acute (during MRgSBRT) GU toxicity, and one (4%) had grade 2 GI toxicity of rectal pain at the completion of MRgSBRT. However, they did not report the results of follow-up outcomes [23]. The researchers from the same group also reported the preliminary outcome results of 1.5T MRgSBRT for oligometastatic castration-sensitive prostate cancer [24,25].

Besides the above-mentioned single-center studies, the initial experience within the MOMENTUM Study (NCT04075305), a prospective international registry of the 1.5T MR-Linac Consortium, was recently reported [25]. The MOMENTUM Study represents the largest multi-center 1.5T MRgRT study so far, with a total patient number of 943, including 281 patients indicated for prostate MRgRT. However, the clinical outcomes of the 281 patients set to receive prostate MRgRT have not yet been published by van Otterloo et al. [25].

The patients in this study were not included in the patient cohort of the MOMENTUM study. Our study observed 11.8% and 2.0% clinician-reported acute grade ≥ 2 GU and GI toxicities, respectively, which were similar to those reported in Alongi et al. [23], but lower than those in the 0.35T studies [20,21,22]. With longer follow-up, our study showed grade ≥ 2 GU and GI toxicities of 2.3% each. These low incidences were mostly consistent with other reports [21,22]. While no grade 3 toxicity was identified in the other studies, two grade 3 GI toxicities, one acute (proctitis) and one subacute (rectal hemorrhage), were observed in a single patient in our study.

Our PROM results were also consistent with those in Tetar et al. [21] in that the effect of MRgSBRT on GU and GI symptoms was most pronounced ~4–6 weeks after treatment, although different PROMs were used (EPIC vs. the European Organization for Research and Treatment of Cancer Quality of Life Questionnaire and International Prostate Symptom Score in [21]). On later follow-ups, urinary and bowel symptoms decreased and returned to baseline levels. The sexual domain summary scores during the follow-ups were generally lower than those at baseline, also similar to the trend presented by Tetar et al. [21]. In both studies, a substantial proportion of patients received ADT, which can greatly affect sexual outcomes, and thus, the impact of MRgSBRT on sexual outcomes could not be clearly differentiated.

Other differences hamper further comparison between our study and Alongi et al.’s 1.5T MRgSBRT study [23]. Our patient cohort (n = 51) included low- to high-risk groups while their study (n = 25) did not include high-risk patients. The two studies also showed some differences in MRgSBRT treatment planning and delivery. Alongi et al.’s SBRT schedule consisted of 5 × 7 Gy fractions delivered on consecutive days, in contrast to our 5 × 7.25 (or 8) Gy twice-per-week fractions. Furthermore, concomitant pelvic nodal radiation (5 × 5 Gy) was given to high-risk patients in our study. For high-risk patients, the benefit of MRI guidance is postulated to be even more substantial to concomitant prostate and pelvic SBRT than to SBRT of the prostate alone. This is because both the target volume and organs at risk (OARs; especially the bowels) are more extensive in the former, and therefore, more adaptation is required for better target coverage and OAR sparing. Regarding online plan adaptation, Alongi et al. applied ATS (100%) to all fractions and our study mostly used ATP (88.6%). They did not describe the use of rectal balloons or rectal spacers. Only three GU and one GI AEs were reported for CROM assessment in Alongi et al., while our more extensive assessment identified five GU and nine GI AEs. We adopted the full EPIC Questionnaire to address PROM-based outcomes, in contrast to the combination of short EPIC-26 and other questionnaires used by Bruynzeel et al. [20]. In contrast to our longitudinal follow-up of up to ~1 year, clinical outcomes were reported at the end of MRgRT without further follow-up by Alongi et al. [23].

It is noteworthy that the patients in this study were enrolled during the pandemic of coronavirus disease 2019 (COVID-19). No patient in this study became infected with severe acute respiratory syndrome coronavirus 2 (SARS-CoV2) during their entire treatment course or follow-up. Additionally, neither treatment nor follow-up were affected by the special management considerations in the era of COVID-19 [26].

This study had some limitations. It was a single-arm observational study rather than a clinical trial with a control group. It comprised a small patient group from a single center, with a short follow-up time. Although we did not use race as either an inclusion or exclusion criterion for patient recruitment, all patients included in this study happened to be of Chinese ethnicity. The clinical outcomes should be considered preliminary. Despite the promising short-term outcome of favorably low GI and GU toxicity, we recognize that long-term outcome results must be observed. Due to the small sample size, correlations between outcomes and patient characteristics and treatment factors were not analyzed. The patient characteristics, treatment factors, and follow-up times were heterogeneous. For example, the substantial proportion of patients that received ADT precluded a reliable assessment of the impact of MRgRT on sexual outcomes. Another notable limitation was PROM assessment based on the single EPIC Questionnaire, rather than a comprehensive set of PROM tools.

## 4. Materials and Methods

### 4.1. Patient Selection

This single-arm observational study was approved by the Hong Kong Sanatorium and Hospital research committee. Written consent was obtained from each patient. Patient inclusion criteria were as follows: age > 18 years; biopsy-proven localized PC, clinical stage T1–T3; no MRI contraindication; no evidence of lymph node metastases or distant metastases on recent diagnostic imaging. Exclusion criteria were as follows: MRI contraindications; previous prostate surgeries or irradiation, or history of other cancers; lack of follow-up CROM or PROM data. MRgSBRT treatment was determined for each eligible patient by both patient choice and multidisciplinary discussion among their urologists, radiation oncologists, radiologists, and medical physicists. Six-month and 18-month regimens of ADT were recommended in unfavorable intermediate-risk and high-risk patients, respectively. ADT was initiated before the commencement of MRgSBRT. Trans-perineal insertion of rectal spacer was optional, arranged ≥2 weeks before RT planning.

### 4.2. Simulation Scan and Treatment Planning

All patients received their CT and MRI simulation scan on the same day (interval ~15–60 min), both in the MRgRT treatment position with a 50–90 mL saline-inflated rectal balloon (QLRAD, Miami, FL, USA). Institutional bladder control protocol was applied to CT and MRI simulation as well as subsequent MRgRT fractions. MRI simulation scan was conducted on a 1.5T MR-simulator (Ingenia MR-RT, Philips Healthcare) using a 3D T2-weighted turbo-spin-echo (3D-T2W-TSE) sequence with almost identical imaging parameters to those used for daily online plan adaptation on the 1.5T MR-Linac (Unity, Elekta, Stockholm, Sweden).

**Figure 3:**
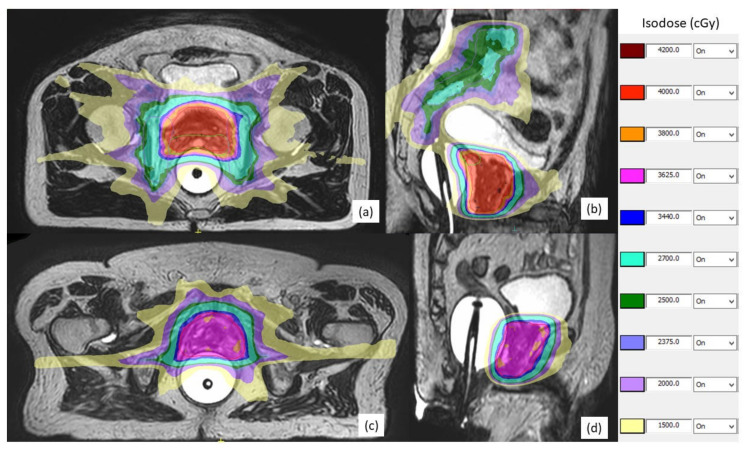
Examples of magnetic resonance-guided stereotactic body radiotherapy plan dose prescription of 40 Gy on a high-risk localized prostate cancer patient (**a**,**b**), and of 36.25 Gy on a low-risk (**c**,**d**) patient. A 25 Gy dose was concurrently prescribed to the pelvic nodal volume for the high-risk patient. Colors are used to illustrate different isodose levels.

Tissue contouring and image registration was conducted using MIM v. 6.9.3 (MIM Software Inc., Cleveland, OH, USA). The clinical target volume (CTV) was contoured by the radiation oncologist on the planning MRI, rigidly registered to the planning CT and, then, propagated to the planning CT images. For low-risk patients, the CTV consisted of the entire prostate gland, while for intermediate- and high-risk patients, the CTV consisted of the prostate gland plus the base of the seminal vesicles. For cT3b cases, the entire seminal vesicles were included in the CTV. Pelvic nodal CTV was delineated starting at the L4–5 junction to include bilateral common/external/internal iliac, presacral, and obturator nodes, as per guidelines by the Radiation Therapy Oncology Group [27]. Planning target volume (PTV) was generated by the isotropic expansion of CTV by 5 mm, except for the 3 mm in the posterior direction for prostate/seminal vesicles. Besides the CTV and PTV, the OARs of the rectum, bladder, bowel, penis, penile bulb, femoral heads, and cauda equina were contoured by radiation dosimetrists following the institutional contouring guidelines. Intensity-modulated RT plans with a mean of 15 beams were generated using Monaco v. 5.40 (Elekta, Stockholm, Sweden). Details of planning objectives and dose constraints of targets and OARs are summarized in Supplementary Table S1. Regarding the dose prescription, 36.25 Gy total dose was prescribed to the whole prostate for low- to intermediate-risk patients and 40 Gy for high-risk patients. For low- to intermediate-risk patients with an intraprostatic dominant lesion (IDL) visible on MRI, 40 Gy was prescribed to the IDL. For high-risk patients, 25 Gy was concurrently prescribed to the pelvic nodal volume. Total dose was delivered in 5 fractions, 2 fractions/week, and completed within 3 weeks. Figure 3 shows examples of plan dose prescription on a high-risk Figure 3a,b and a low-risk Figure 3c,d patient.

### 4.3. Treatment Delivery and Adaptation

At each fraction, patients were positioned before undergoing a daily MRI scan on the 1.5T MR-Linac to obtain on-the-date anatomy information to facilitate online plan adaptation, using either adapt-to-position (ATP) and adapt-to-shape (ATS) workflow [16]. The ATP workflow was the default, used with higher priority than ATS to minimize treatment duration and to compensate for the isocenter shift, since the use of rectal balloon helped to reduce intra-/inter-fractional prostate motion. The ATS workflow was only selected when encountering a severe change in body contour, target/OAR shape, or relative position change between the target and OARs. In the ATS workflow, the attending oncologist adapted the contours of the CTV, rectum, bladder, and bowel through a deformable registration and/or manual adjustment. This was followed by re-optimization of the MRI-based treatment plan. Optimal target coverage was prioritized while restricting the high dose to OARs.

### 4.4. Patient Follow-Up and Outcome Measurements

The primary clinical endpoint was clinical outcome based on both CROM and PROM. CROM includes the assessment of GI and GU adverse events by the radiation oncologist, based on the Common Terminology Criteria for Adverse Events Scale v. 5.0, at baseline, weekly during MRgSBRT, 1 month, and every 3 months thereafter. GU AEs, including urinary frequency, urinary incontinence, urinary retention, urinary tract pain, and urinary urgency, and GI AEs, including abdominal pain, bloating, constipation, diarrhea, fecal incontinence, nausea, proctitis, rectal hemorrhage, and rectal pain, were assessed.

EPIC, a well-validated domain-specific patient-reported questionnaire, was used for PROM assessment [28]. EPIC assesses QoL in four domains of urinary function (7 questions with 12 items), bowel habits (9 questions with 14 items), sexual function (9 questions with 13 items), and hormonal function (6 questions with 11 items). PROM data were collected at the pre-treatment baseline, 1 month after completion of MRgRT, and then, at follow-ups about every 3 months.

Prostate-specific antigen (PSA) levels at baseline and each follow-up were also collected.

### 4.5. Statistical Analysis

Descriptive statistics are presented as mean ± standard deviation. Incidences of acute (from the beginning of MRgSBRT until ≤30 days after the completion of RT) and subacute (>30 days after the completion of MRgSBRT) GU and GI AEs were calculated. EPIC Questionnaires were scored on a scale of 0 to 100 according to the standardized scoring instructions [28]. Higher scores indicated better QoL. Longitudinal assessment of EPIC-derived domain summary scores and domain-specific subscale scores at each time point was conducted using repeated ANOVA measures) or the Mann–Whitney U test, where appropriate. All statistical analyses were performed using R v1.2 (RStudio, Boston, MA, USA).

## 5. Conclusions

This prospective single-center study is the first report of preliminary longitudinal clinical outcomes of 1.5T MRgSBRT in localized PC based on both CROM and PROM, featuring follow-ups of up to 1 year. Low incidences of clinician-reported grade ≥ 2 GI and GU AEs were observed. Patient-reported urinary, bowel, and hormonal domain summary EPIC scores dropped at 1 month and, then, gradually returned to baseline levels. These findings, despite being preliminary, support the continued assessment of the clinical value of 1.5T MRgSBRT in localized PC.

## Figures and Tables

**Table 1:** Patient characteristics at baseline (N = 51).

Characteristics	Number of Patients	Percentage
Age (years)		
Mean ± SD	71.5 ± 7.7	
Range	56–90	
ECOG performance status		
0	49	96.1%
1	2	3.9%
Prostate volume (cc)		
Mean ± SD	47.04 ± 32.33	
Clinical T Stage		
1	4	7.8%
2	38	74.5%
3	9	17.6%
	ECE presence	
No	42	82.4%
Yes	9	17.6%
Pre-MRgSBRT PSA (ng/mL)		
<10	28	54.9%
10–20	13	25.5%
>20	10	19.6%
Median PSA	8.98	
Gleason score (ISUP Prostate Cancer Grade Group)		
3 + 3 (Grade 1)	18	35.3%
3 + 4 (Grade 2)	20	39.2%
4 + 3 (Grade 3)	5	9.8%
4 + 4 (Grade 4)	4	7.8%
4 + 5 (Grade 5)	4	7.8%
Risk classification (NCCN)		
Low	4	7.8%
Intermediate	29	56.9%
High	18	35.3%
ADT prescription		
No	21	41.2%
Yes	30	58.8%
Rectal spacer		
Yes	10	19.6%
No	41	80.4%

SD = standard deviation; ECOG = Eastern Cooperative Oncology Group; ECE = extracapsular extension; MRgSBRT = magnetic resonance-guided stereotactic body radiotherapy; PSA = prostate-specific antigen; ISUP = International Society of Urological Pathology; NCCN = National Comprehensive Cancer Network; ADT = androgen-deprivation therapy.

**Table 2:** Incidences of clinician-reported gastrointestinal (GI) and genitourinary (GU) adverse effects based on the Common Terminology Criteria for Adverse Events Scale (CTCAE) v. 5.0 in the patients.

Incidence	Grade 0	Grade 1	Grade 2	Grade 3	Grade ≥ 2
GI Adverse Effects					
Baseline (before MRgSBRT)	98.0% (n = 50)	2.0% (n = 1)	0	0	0
(N = 51)					
Acute (during and ≤30 days after MRgSBRT)	78.4%	19.6%	0	2.0%	2.0%
(N = 51)	(n = 40)	(n = 10)	(n = 0)	(n = 1)	(n = 1)
Subacute (>30 days after MRgSBRT)	88.4%	9.3%	0	2.3%	2.3%
(N = 43)	(n = 38)	(n = 4)		(n = 1)	(n = 1)
**GU Adverse Effects**					
Baseline (before MRgSBRT)	52.9%	47.1%	0	0	0
(N = 51)	(n = 27)	(n = 24)			
Acute (during and ≤30 days after MRgSBRT)	11.8%	76.5%	11.8%	0	11.8%
(N = 51)	(n = 6)	(n = 39)	(n = 6)		(n = 6)
Subacute (>30 days after MRgSBRT)	39.5%	58.1%	2.3%	0	2.3%
(N = 43)	(n = 17)	(n = 25)	(n = 1)		(n = 1)

MRgSBRT = magnetic resonance-guided stereotactic body radiotherapy.

**Table 3:** Patient-reported outcome measurements based on the Expanded Prostate Cancer Index Composite (EPIC) Questionnaire.

EPIC Score	Follow-Up Time Points						One-Way ANOVA *p*-Value
	Baseline	1 Month	4 Months	7 Months	10 Months	13 Months	
Patients (n)	51	51	39	29	14	5	
**Domain Summary Scores**							
Urinary	84.98 ± 13.40	76.75 ± 18.25	84.22 ± 12.42	88.98 ± 12.60	91.82 ± 11.14	91.95 ± 6.44	**0.0028**
Bowel	91.62 ± 10.31	85.58 ± 14.44	90.38 ± 10.88	91.53 ± 10.62	91.21 ± 8.61	91.79 ± 5.73	0.2112
Sexual	34.79 ± 18.00	30.92 ± 20.48	25.89 ± 16.03	26.68 ± 18.98	28.90 ± 16.50	35.30 ± 20.83	0.3373
Hormonal	89.18 ± 12.40	86.44 ± 14.13	87.51 ± 13.25	87.04 ± 11.24	88.64 ± 11.29	94.32 ± 8.40	0.8368
**Domain-Specific Subscales**							
**Urinary Subscales**							
Function	94.91 ± 9.84	85.98 ± 13.97	92.86 ± 8.21	95.83 ± 5.56	96.44 ± 5.56	97.68 ± 5.19	**0.0003**
Bother	79.09 ± 18.40	70.70 ± 22.93	78.34 ± 18.99	88.52 ± 18.22	88.52 ± 18.22	87.86 ± 7.41	**0.0298**
Incontinence	88.49 ± 13.32	82.99 ± 17.68	86.91 ± 14.63	92.26 ± 11.10	92.88 ± 9.84	97.10 ± 6.48	0.0515
Irritative/Obstructive	82.94 ± 15.11	73.78 ± 20.61	82.20 ± 15.10	86.83 ± 14.87	90.82 ± 15.26	89.29 ± 4.37	**0.0072**
**Bowel Subscales**							
Function	91.67 ± 7.17	86.77 ± 11.02	89.98 ± 9.35	92.24 ± 9.45	92.03 ± 6.04	92.14 ± 6.87	0.0933
Bother	90.37 ± 18.09	84.29 ± 20.79	88.82 ± 19.02	90.87 ± 14.30	91.07 ± 16.97	91.43 ± 5.98	0.6436
**Sexual Subscales**							
Function	23.64 ± 19.20	19.48 ± 21.45	13.71 ± 17.76	13.45 ± 17.51	15.03 ± 19.92	32.86 ± 17.67	0.0904
Bother	61.40 ± 34.21	60.64 ± 37.81	58.27 ± 31.52	57.45 ± 35.75	57.29 ± 26.09	53.75 ± 32.05	0.9934
**Hormonal Subscales**							
Function	86.84 ± 16.18	84.64 ± 16.90	87.57 ± 11.57	86.61 ± 10.81	88.46 ± 12.65	92.50 ± 11.90	0.8651
Bother	90.72 ± 14.25	87.03 ± 20.20	87.12 ± 19.81	87.50 ± 16.55	88.78 ± 17.71	95.83 ± 5.89	0.8754

One-way analysis of variance (ANOVA) *p*-values are calculated based on all time points. Number in bold indicates statistical significance.

## Data Availability

The data presented in this study are available on request from the corresponding author. The data are not publicly available to comply with our hospital policy on patient privacy protection.

## Supplementary Materials

The following are available online at https://www.mdpi.com/article/10.3390/cancers13194866/s1, Table S1: Planning objectives and dose constraints for organs at risk (OARs).
