# Supplementary material for: 1.5T Magnetic Resonance-Guided Stereotactic Body Radiotherapy for Localized Prostate Cancer: Preliminary Clinical Results of Clinician- and Patient-Reported Outcomes
Source: Cancers (Basel). 2021 Sep 28;13(19):4866. doi: 10.3390/cancers13194866 (PMC8508440; doi:10.3390/cancers13194866)
Supplement: Supplementary file 1 [file cancers-13-04866-s001.zip › cancers-1375645-supplementary.pdf]

Table S1. Planning objectives and dose constraints for organs-at-risk (OARs).

Structure	Low- and intermediate-risk	High-risk
	**Planning objectives**	
PTV	V38.8 Gy < 0.03cc<br>V36.25 Gy > 95%	V42 Gy < 0.03cc<br>V40 Gy > 95%
	**OAR dose constraints**	
Rectum	V38.1 Gy < 0.03cc<br>V34.4 Gy < 3cc<br>V32.6 Gy < 10%<br>V29 Gy < 20%<br>V18.1 Gy < 50%	V41.6 Gy < 0.03cc<br>V38 Gy < 3cc<br>V36 Gy < 10%<br>V32 Gy < 20%<br>V20 Gy < 50%
Bladder	V38.1 Gy < 0.03cc<br>V32.6 Gy < 10%<br>V18.1 Gy < 50%	V41.6 Gy < 1cc<br>V37 Gy < 5cc<br>V36 Gy < 10%<br>V20 Gy < 50%<br>V18.1 Gy < 60%
Femoral Head	V20 Gy < 10cc<br>Dmax < 30 Gy	V20 Gy < 10cc<br>Dmax < 30 Gy
Penile Bulb	V20 Gy < 3cc<br>Dmax < 36.25 Gy	D2% < 28.5 Gy<br>V20 Gy < 3cc<br>Dmax < 40 Gy<br>Dmean < 16 Gy

Dmax/Dmean = maximum/mean dose; PTV = planning target volume.
